# Lung Adenocarcinoma Mouse Models Based on Orthotopic Transplantation of Syngeneic Tumor-Initiating Cells Expressing EpCAM, SCA-1, and Ly6d

**DOI:** 10.3390/cancers12123805

**Published:** 2020-12-17

**Authors:** Takashi Semba, Ryo Sato, Akiyoshi Kasuga, Kentaro Suina, Tatsuhiro Shibata, Takashi Kohno, Makoto Suzuki, Hideyuki Saya, Yoshimi Arima

**Affiliations:** 1 Division of Gene Regulation, Institute for Advanced Medical Research, Keio University School of Medicine, 35 Shinano-machi, Shinjuku-ku, Tokyo 160-8582, Japan; TSemba1@mdanderson.org (T.S.); sator3@nih.gov (R.S.); akiyoshi.kasuga@jfcr.or.jp (A.K.); ksuina@keio.jp (K.S.); hsaya@a5.keio.jp (H.S.); 2 Department of Thoracic Surgery, Kumamoto University, 1-1-1 Honjo, Chuo-ku, Kumamoto 860-8556, Japan; smakoto@kumamoto-u.ac.jp; 3 Department of Respiratory Medicine, Kumamoto University, 1-1-1 Honjo, Chuo-ku, Kumamoto 860-8556, Japan; 4 Division of Gastroenterology and Hepatology, Department of Internal Medicine, Keio University School of Medicine, 35 Shinanomachi, Shinjuku-ku, Tokyo 160-8582, Japan; 5 Division of Cancer Genomics, National Cancer Center Research Institute, 1-1, Tsukiji 5-chome, Chuo-ku, Tokyo 104-0045, Japan; tashibat@ncc.go.jp; 6 Division of Genome Biology, National Cancer Center Research Institute, 1-1, Tsukiji 5-chome, Chuo-ku, Tokyo 104-0045, Japan; tkkohno@ncc.go.jp

**Keywords:** lung cancer, syngeneic mouse model, orthotopic transplantation, EpCAM, SCA-1, Ly6d

## Abstract

**Simple Summary:**

Lung cancer is the leading cause of cancer deaths worldwide and lung adenocarcinoma (LUAD) is the most common type of lung cancer. To better understand the relation between genetic alterations and the characteristics of lung cancer as well as the interactions between tumor cells and components of the tumor microenvironment, we have developed organoid-based orthotopic and syngeneic mouse models of LUAD driven by the *KRAS*^G12V^ or *EML4-ALK* oncogene. These models formed tumors closely recapitulating the pathology of human LUAD and proved useful tools for in vitro and in vivo drug efficacy studies. In addition, with the use of these models, we identified Ly6d as a potential novel cancer stem cell marker for LUAD. Given their clinical relevance, our mouse models are important tools for studying cancer stem cell biology and LUAD drug development.

**Abstract:**

Somatic mutations in *EGFR* and *KRAS* as well as chromosome rearrangements affecting *ALK*, *ROS1*, and *RET* have been identified in human lung adenocarcinoma (LUAD). We here developed organoid-based orthotopic and syngeneic mouse models for studies of the pathogenesis and treatment of LUAD. We isolated EpCAM-positive epithelial cells from mouse lungs and cultured them as organoids to maintain epithelial stem cell properties. These cells were transformed by KRAS(G12V) or EML4-ALK and then transplanted via the trachea into the lungs of the syngeneic mice, where they formed tumors that expressed the lung lineage marker TTF-1 and which closely recapitulated the pathology of human LUAD. Treatment with crizotinib suppressed the growth of tumors formed by the EML4-ALK–expressing lung epithelial cells in a subcutaneous transplantation model. Organoid culture of normal lung epithelial cells resulted in enrichment of EpCAM^+^SCA-1(Ly6a)^+^ cells as well as in that of cells expressing another member of the Ly6 protein family, Ly6d, which was found to be required for the growth of the LUAD-initiating cells expressing KRAS(G12V) or EML4-ALK. We also found that a high expression level of *LY6D* was associated with poor prognosis in human LUAD. Our results thus suggest that LY6D is a potential lung cancer stem cell marker.

## 1. Introduction

Lung cancer is one of the most frequently diagnosed cancers and the leading cause of cancer deaths worldwide [1]. Non–small cell lung cancer (NSCLC)—including adenocarcinoma, squamous cell carcinoma, and large cell carcinoma—accounts for ~85% of all lung cancer cases [2], with lung adenocarcinoma (LUAD) being the most prevalent type of NSCLC [3]. Recent genome-wide surveys have revealed various gene alterations associated with LUAD, including mutations of the epidermal growth factor receptor (*EGFR*) and Kristen rat sarcoma viral oncogene homolog (*KRAS*) genes as well as chromosome rearrangements involving the anaplastic lymphoma kinase gene (*ALK*) [4,5,6,7]. Although molecularly targeted therapies have markedly improved treatment outcome for individuals with LUAD driven by mutant forms of *EGFR* [8] or by *ALK* fusion genes [9], the contributions of these and other genetic changes to the characteristics of LUAD and the molecular mechanisms of resistance to such targeted therapies remain to be addressed [10].

Interactions between tumor cells and components of the tumor microenvironment (TME)—such as stromal cells, endothelial cells, and immune cells—play key roles in tumor initiation and progression including in lung cancer [11,12]. It is therefore imperative to develop appropriate cancer models that will provide insight into the pathogenesis and treatment of LUAD. Many xenograft models based on injection of mice with human lung cancer cell lines as well as various genetically engineered mouse models (GEMMs) have been established [13,14]. However, xenograft models that rely on immunodeficient mice (nude or SCID mice) are not able to fully recapitulate the TME. Although GEMMs are suitable for investigation of whether a certain gene alteration induces tumor formation or whether an altered gene functions as an oncogene or a tumor suppressor gene, the generation and maintenance of such models are time-consuming and require a considerable amount of effort. In addition, parameters such as the incidence of tumorigenesis, timing of tumor initiation, and course of tumor progression show more variation among animals in the case of GEMMs compared with transplantation models [15]. On the other hand, murine lung cancer cell lines such as Lewis lung carcinoma (LLC) cells can be injected into syngeneic mice to establish immunocompetent and orthotopic mouse models that recapitulate the lung TME [13]. However, types of murine lung cancer cell lines to be used for such purpose are limited and are not suitable for investigation of the contribution of oncogenic gene alterations to the characteristics of LUAD.

We have previously generated a series of syngeneic mouse models for many types of malignancy, including osteosarcoma [16], leukemia-lymphoma [17], glioblastoma [18], choriocarcinoma [19], phyllodes tumors [20], ovarian cancer [21], and biliary tract cancer [22]. In these models, tissue-specific stem or progenitor cells of adult mice are transformed into tumor-initiating cells by gene modifications, and transfer of the resulting cells to syngeneic mice leads to the formation of tumors that recapitulate the phenotype of corresponding human malignancies. We have now established syngeneic and orthotopic mouse models of LUAD with this approach—specifically, by deletion of *Cdkn2a* and introduction of a mutant form of *KRAS* or an *EML4-ALK* fusion gene. Moreover, these models revealed Ly6d to be a potential novel cancer stem cell (CSC) marker for LUAD. Our models thus have the potential to shed light on the pathophysiology of LUAD as well as to inform the development of new therapeutic strategies.

## 2. Results

### 2.1. Organoid Culture of Murine Lung Epithelial Cells

**Figure 1:**
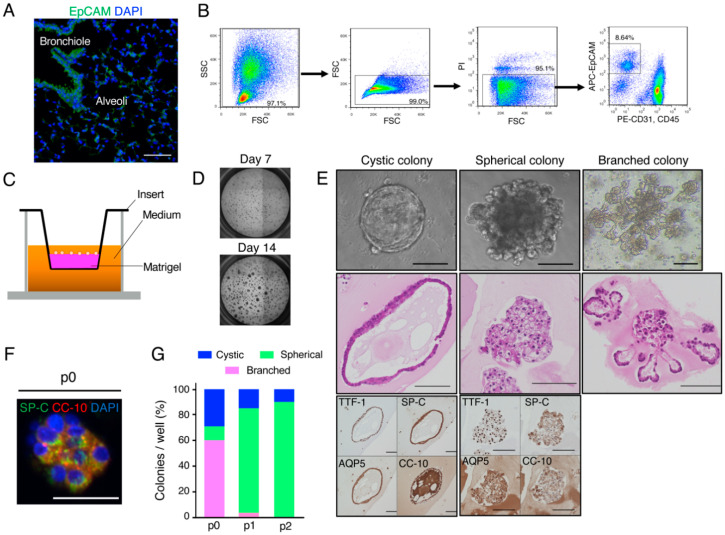
Three-dimensional culture of mouse lung epithelial cells. (**A**) Representative immunofluorescence staining of epithelial cells in a mouse lung section with antibodies to EpCAM. Nuclei were stained with 4′,6-diamidino-2-phenylindole (DAPI). Scale bar, 100 µm. (**B**) Gating strategy for sorting of EpCAM^+^CD31^−^CD45^−^ lung epithelial cells by FACS. SSC, side scatter; FSC, forward scatter; PI, propidium iodide; APC, allophycocyanin; PE, phycoerythrin. (**C**) Schematic representation of the 3D culture system. (**D**) Representative phase-contrast images of primary EpCAM^+^CD31^−^CD45^−^ lung epithelial cells expanded in 3D culture. (**E**) Representative phase-contrast microscopy (upper row), hematoxylin-eosin staining (middle row), and immunocytochemical staining of TTF-1, SP-C, AQP5, or CC-10 (lower row) for colonies of lung epithelial cells. Scale bars, 100 µm. Three types of colony morphology—cystic, spherical, and branched—were apparent. (**F**) Representative fluorescent multiplex immunostaining of SP-C and CC-10 in a primary (p0) lung epithelial cell colony. Nuclei were stained with DAPI. Scale bar, 50 µm. (**G**) Quantification of colony types for lung epithelial cells before (p0) and after consecutive serial passages (p1, p2). Data are means of triplicates from a representative experiment.

We first isolated mouse lung epithelial cells that express epithelial cell adhesion molecule (EpCAM) (Figure 1A). EpCAM^+^CD31^−^CD45^−^ epithelial cells were thus isolated from adult C57BL/6 mouse lungs by fluorescence-activated cell sorting (FACS) (Figure 1B). The sorted cells formed colonies (or organoids) within 7 to 14 days in a three-dimensional (3D) culture system including Matrigel and serum-free medium supplemented with EGF, keratinocyte growth factor (KGF), and a Rho-kinase (ROCK) inhibitor (Figure 1C,D). These colonies showed three different types of morphology: (1) cystic colonies, which in cross-section resembled bronchial cavity–like structures; (2) spherical colonies that were densely packed with cells; and (3) large and branched colonies whose histology resembled that of alveoli (Figure 1E). Immunocytochemical analysis revealed that the cells in these colonies all expressed thyroid transcription factor–1 (TTF-1), a lung lineage marker, and many of the cells also expressed differentiation markers such as aquaporin 5 (AQP5), surfactant protein–C (SP-C), or Clara cells 10-kDa secretory protein (CC-10) (Figure 1E). We also detected the presence of bronchioalveolar stem cells (BASCs) [23,24,25], a lung stem cell population that expresses both SP-C and CC-10, in the colonies (Figure 1F). The lung epithelial cells had the potential to be cultured for more than four passages, but sphere-type colonies predominated after the first passage (Figure 1G).

### 2.2. Expansion of Oncogene-Transformed Lung Epithelial Cells Supported by Cdkn2a Knockout

**Figure 2:**
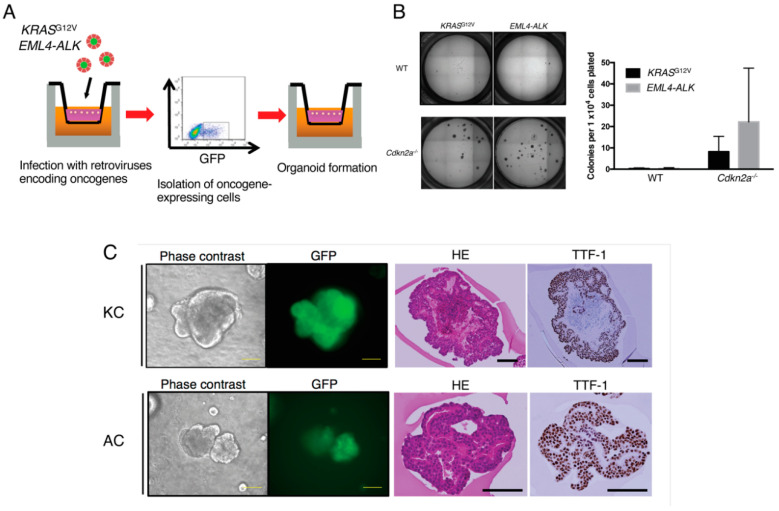
Expansion of oncogene-transformed mouse lung epithelial cells supported by *Cdkn2a* knockout. (**A**) Schematic representation of oncogene transduction in lung epithelial cells. Cells dissociated from colonies in 3D culture were infected with retroviruses harboring GFP and either *KRAS*^G12V^ or *EML4-ALK* cDNAs, after which GFP-positive cells were isolated by FACS and subjected to organoid culture. (**B**) Colony formation efficiency for lung epithelial cells from wild-type (WT) or *Cdkn2a*^−/−^ mice after retroviral transduction of *KRAS*^G12V^ or *EML4-ALK*. Data are means + SD (*n* = 3 independent experiments). Representative phase-contrast images of culture plates are also shown. (**C**) Representative phase-contrast and fluorescence microscopy as well as hematoxylin-eosin (HE) and TTF-1 immunocytochemical staining of colonies formed by KC cells or AC cells. Scale bars, 100 µm.

We next introduced either the G12V mutant form of *KRAS* or *EML4-ALK*, both of which are oncogenes for human LUAD, into cultured mouse lung epithelial cells. The colonies formed in 3D cultures of lung epithelial cells were thus dissociated into single cells, which were then infected with retroviruses harboring the cDNA for green fluorescent protein (GFP) linked via an internal ribosomal entry sequence (IRES) to either *KRAS*^G12V^ or *EML4-ALK* cDNA. The GFP-positive cells were then isolated by FACS and again subjected to 3D culture (Figure 2A). However, we found that the cells did not proliferate after forced oncogene expression (Figure 2B), consistent with previous observations that transformation of primary cells by oncogenes induces cellular senescence [26,27].

The cyclin-dependent kinase inhibitor 2A (CDKN2A) gene locus encodes two tumor suppressor proteins, p16^Ink4a^ and Arf, that are key inducers of cellular senescence associated with both aging and cancer [28]. Given that somatic inactivation of the *CDKN2A* locus by deletion or methylation is common in LUAD [5,29], we tested whether *Cdkn2a* knockout in mouse lung epithelial cells might confer resistance to oncogene-induced senescence. We found that EpCAM^+^CD31^−^CD45^−^ lung epithelial cells isolated from *Cdkn2a*^−/−^ mice [27] underwent continuous proliferation even after introduction of either *KRAS*^G12V^ or *EML4-ALK* (Figure 2B). Both *KRAS*^G12V^-transformed *Cdkn2a*^−/−^ lung epithelial cells (hereafter referred to as KC cells) and *EML4-ALK*–transformed *Cdkn2a*^−/−^ lung epithelial cells (AC cells) formed morphologically distorted colonies in 3D culture (Figure 2C). Immunoblot analysis confirmed the expression of exogenously introduced KRAS and ALK in both the KC and AC cells, respectively (Supplementary Figure S1). Although the organoids formed by KC or AC cells contained necrotic debris and showed disruption of cell alignment, TTF-1 expression was maintained (Figure 2C), suggesting that the cells had acquired atypical proliferative capacity as a result of oncogene transduction while retaining their lung cell properties.

### 2.3. Establishment of Clinically Relevant Syngeneic Mouse Models of LUAD

**Figure 3:**
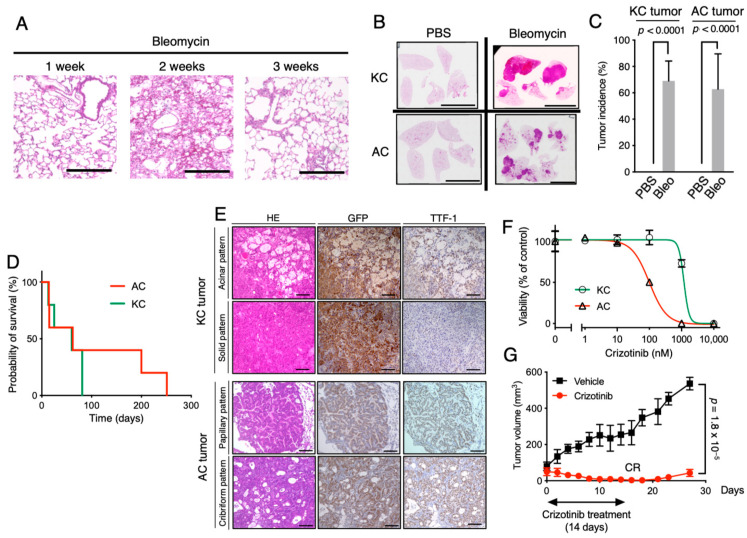
Establishment of clinically relevant syngeneic mouse models of LUAD. (**A**) Representative hematoxylin-eosin staining of the lungs of C57BL/6 mice at 1, 2, and 3 weeks after intratracheal bleomycin administration. Scale bars, 100 µm. (**B**) Representative hematoxylin-eosin staining of the lungs of PBS- or bleomycin-pretreated mice at 28 days after intratracheal transfer of KC or AC cells. Scale bars, 1 cm. (**C**) Lung tumor incidence in PBS- or bleomycin-pretreated mice at 28 days after KC or AC cell transfer. Data are means + SD (*n* = 5 recipient mice). The *p* values were calculated with the two-tailed Student’s *t* test. (**D**) Kaplan-Meier survival curves for KC or AC lung tumor–bearing mice (*n* = 5 for each model) from the time of cell transfer. (**E**) Representative hematoxylin-eosin staining as well as immunohistochemical staining of GFP or TTF-1 in lung tumors isolated 28 days after KC or AC cell transfer. Scale bars, 100 μm. (**F**) Viability of KC or AC cells cultured in the presence of the indicated concentrations of crizotinib for 72 h. Data are means ± SD of triplicates from a representative experiment. (**G**) Nude mice bearing subcutaneous tumors formed by injected AC cells were treated with vehicle or crizotinib for 14 days, and tumor volume was determined at the indicated times after the onset of treatment. CR indicates complete tumor regression. Data are means ± SD (*n* = 5 mice), and the *p* value was calculated with the two-tailed Student’s *t* test.

We tried to determine if KC/AC cells could form tumors in syngeneic mouse lungs. Intratracheal transfer of mouse lung cancer cells, such as LLC cells, has previously been shown to result in the formation of orthotopic lung tumors in syngeneic mice [30,31]. However, transfer of either KC or AC cells via the trachea did not give rise to tumors in the lungs of C57BL/6 mice. We previously showed that bleomycin-induced lung fibrosis promotes metastatic tumor cell colonization in the lung [32], with such lung fibrosis having been found to persist for only a relatively short period of time [33]. We therefore examined whether transient bleomycin-induced fibrosis might promote AC or KC cell colonization and tumor formation in the lung. We first confirmed that lung fibrosis induced by intratracheal bleomycin administration in C57BL/6 mice was prominent at 2 weeks but had resolved by 3 weeks after bleomycin treatment (Figure 3A).

We then transferred KC or AC cells into the lungs of C57BL/6 mice via the trachea at 2 weeks after bleomycin administration. Both KC and AC cells formed large tumor nodules that were apparent 4 weeks after cell transfer in bleomycin-pretreated recipients, with a tumor incidence of 69% and 63%, respectively, whereas neither cell type gave rise to tumors in mice pretreated with phosphate-buffered saline (PBS) as vehicle (Figure 3B,C). The median survival time of bleomycin-pretreated mice bearing KC or AC tumors was 62 and 60 days, respectively (Figure 3D). Histopathologic analysis revealed that both KC and AC cells formed well-differentiated adenocarcinoma in the lungs of recipient mice with pathomorphological similarities to human LUAD (Figure 3E). The GFP^+^ tumor cells in both KC and AC models expressed TTF-1, a clinically established LUAD marker. No obvious fibrotic foci were observed in the lungs in either the KC or AC model (data not shown). In the KC model, acinar- and solid-type tumors were observed (Figure 3E). In the AC model, papillary-type tumors as well as tumors showing a cribriform pattern that is frequently found in human LUAD positive for the *EML4-ALK* fusion gene were detected (Figure 3E). Together, these observations showed that the KC and AC mouse models recapitulated the pathological phenotypes of human LUAD. The expression of TTF-1 tended to be at a low level in cells of tumor nodules showing the solid pattern in the KC model.

We next treated KC and AC cells with crizotinib, an ALK inhibitor administered clinically in patients with LUAD positive for *ALK* fusion genes [34]. Crizotinib inhibited the proliferation of AC and KC cells with a median inhibitory concentration (IC_50_) of 99 and 1187 nM, respectively (Figure 3F), consistent with results obtained with human lung cancer cell lines showing that NCI-H3122 (*EML4-ALK* positive) was much more sensitive to crizotinib than was A549 (*KRAS*^G12S^ positive) [35]. Subcutaneous injection of AC cells in the dorsal flank of nude mice gave rise to palpable tumors at 4 weeks after injection, and we found that treatment of these mice with crizotinib resulted in complete tumor regression after 14 days and that the tumors regrew after crizotinib discontinuation (Figure 3G). These results thus suggested that the proliferation of AC cells is dependent on ALK activity, as is the case for LUAD harboring *ALK* gene rearrangements [36], and that our tumor models might prove useful tools for in vitro and in vivo studies of drug efficacy. Our KC and AC models closely recapitulate human LUAD and are clinically relevant.

### 2.4. Stem Cell Properties of KC and AC Cells and Similar Expression Patterns of SCA-1 (Ly6a) and Ly6d in Mouse LUAD-Initiating Cells

**Figure 4:**
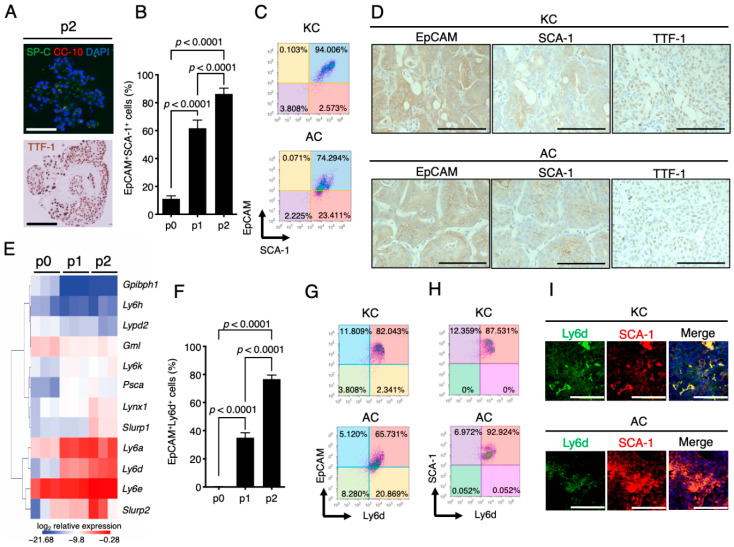
Increased expression of SCA-1 and Ly6d in lung epithelial cells with culture and in oncogene-expressing tumorigenic lung epithelial cells. (**A**) Representative immunofluorescence staining of SP-C and CC-10 (nuclei were stained with DAPI) as well as immunochemical staining of TTF-1 in p2 colonies of mouse lung epithelial cells. Scale bars, 100 µm. (**B**) Flow cytometric analysis of the proportion of EpCAM^+^SCA-1^+^ cells among primary lung epithelial cells (p0) or cells of subsequent serial passages (p1, p2). Data are means + SD of triplicates from a representative experiment, and the *p* values were calculated by one-way analysis of variance (ANOVA) followed by Tukey’s multiple-comparison test. (**C**) Representative flow cytometric analysis of EpCAM and SCA-1 expression on KC or AC cells. (**D**) Representative immunohistochemical staining for EpCAM, SCA-1, or TTF-1 in serial sections of lung tumors from KC and AC mouse models. Scale bars, 100 µm. (**E**) Heatmap showing the changes in mRNA abundance for Ly6/uPAR family genes in mouse lung epithelial cells during serial passage in 3D culture. Data are shown for three independent experiments. (**F**) Flow cytometric analysis of the proportion of EpCAM^+^Ly6d^+^ cells among primary lung epithelial cells (p0) or cells of subsequent serial passages (p1, p2). Data are means + SD of triplicates from a representative experiment, and the *p* values were calculated by one-way ANOVA followed by Tukey’s multiple-comparison test. (**G**,**H**) Representative flow cytometric analysis of EpCAM and Ly6d expression (**G**) and of SCA-1 and Ly6d expression (**H**) on KC or AC cells. (**I**) Representative immunofluorescence staining of KC or AC lung tumor sections for Ly6d and SCA-1. Nuclei were stained with DAPI. Scale bars, 100 µm.

We observed that after the first passage of normal mouse lung epithelial cells in 3D culture, most of the colonies with structures similar to bronchi or alveoli (cystic colonies and branched colonies, respectively) disappeared, with spherical colonies becoming the predominant colony type (Figure 1G). Furthermore, the cells in these latter colonies expressed few differentiation markers, including SP-C and CC-10, but they retained expression of TTF-1 (Figure 4A), suggesting that the proportion of undifferentiated lung cells expanded during culture. Indeed, flow cytometry revealed that the proportion of EpCAM and stem cell antigen–1 (SCA-1) double-positive cells, which constitute a population of mouse lung progenitors [25,37], increased significantly among cultured lung epithelial cells with each passage (Figure 4B). These results thus suggested that lung epithelial stem/progenitor cells were enriched by our culture condition. Flow cytometry revealed that >95% of KC and AC cells were positive for SCA-1 (97% and 98%, respectively) (Figure 4C), with SCA-1^+^ cells being implicated as CSCs that possess a high tumor-propagating ability and which give rise to intra-tumoral heterogeneity through generation of a differentiation hierarchy [38,39,40,41]. Indeed, we detected a heterogeneous pattern of SCA-1 expression in both KC and AC tumors in syngeneic mouse lung (Figure 4D), despite the observation that nearly all KC and AC cells expressed SCA-1 in vitro, suggesting that SCA-1^+^ tumor cells generated SCA-1^−^ tumor cells in vivo and providing further support for the notion that KC and AC cells possess CSC-like properties.

SCA-1 is encoded by *Ly6a* on mouse chromosome 15 and belongs to the Ly6/uPAR family of proteins [42]. Although SCA-1 has been extensively studied as a stem cell marker in mouse, an ortholog of SCA-1 has not been identified in human [43]. Several Ly6/uPAR family genes have been identified on mouse chromosome 15 and have orthologs on the syntenic human chromosome 8, but the functional similarity of these Ly6/uPAR family molecules to SCA-1 remains unclear. We performed hierarchical clustering analysis for the expression of *Ly6a* and 11 other murine Ly6/uPAR family genes that possess human orthologs in primary mouse lung epithelial cells before (p0) and after consecutive serial passages (p1, p2), and we found that the expression pattern of *Ly6d* was highly similar to that of *Ly6a* (Figure 4E). We also observed that, similar to that of SCA-1 (Figure 4B), the cell surface expression of Ly6d on lung epithelial cells increased significantly with each passage (Figure 4F). These results suggested that Ly6d might also function as a stem cell marker in murine lung stem cells, consistent with evidence implicating Ly6d as a marker for several types of adult progenitor cells [44,45,46]. Furthermore, we found that >80% of KC and AC cells were positive for Ly6d (84% and 87%, respectively) (Figure 4G) and that >85% of SCA-1^+^ KC or AC cells also expressed Ly6d (88% and 93%, respectively) (Figure 4H). In addition, multiplex immunofluorescence analysis revealed that SCA-1 and Ly6d were co-expressed in KC and AC lung tumor cells (Figure 4I). Together, these results suggested that Ly6d is a potential marker of CSCs in mouse LUAD.

### 2.5. Ly6d Is Required for Colony Formation by Oncogene-Expressing Lung Epithelial Cells in 3D Culture

**Figure 5:**
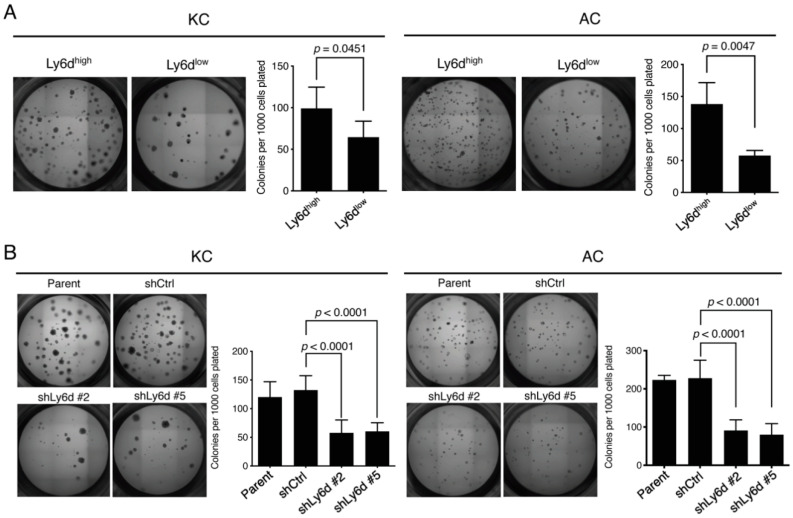
Ly6d is required for colony formation by oncogene-transformed lung epithelial cells in 3D culture. (**A**) Ly6d^high^ and Ly6d^low^ cells isolated by FACS from KC or AC cells were seeded in 3D culture for determination of colony formation efficiency. (**B**) Colony formation in 3D culture by KC or AC cells transfected with expression vectors for control (shCtrl) or Ly6d (shLy6d #2 or #5) shRNAs. All quantitative data are means + SD (*n* = 5), and the *p* values were calculated with the two-tailed Student’s *t* test (**A**) or by one-way ANOVA followed by Tukey’s multiple-comparison test (**B**).

To investigate the function of Ly6d, we isolated KC or AC cells that express the protein at a high level (Ly6d^high^ cells) or a low level (Ly6d^low^ cells) by FACS (Supplementary Figure S2A). We found that Ly6d^high^ KC or AC cells generated significantly more colonies in 3D culture than did the corresponding Ly6d^low^ cells (Figure 5A). Moreover, knockdown of *Ly6d* mRNA with specific short hairpin RNAs (shRNAs) in KC or AC cells resulted in a significant reduction in the number of colonies formed in 3D culture (Figure 5B, Supplementary Figure S2B). These results indicated that Ly6d plays a key role in colony formation by lung tumor cells, and they therefore further suggested that Ly6d is a functional stem cell marker for mouse LUAD.

### 2.6. Expression of Ly6d Is Associated with Poor Prognosis in LUAD Patients

**Figure 6:**
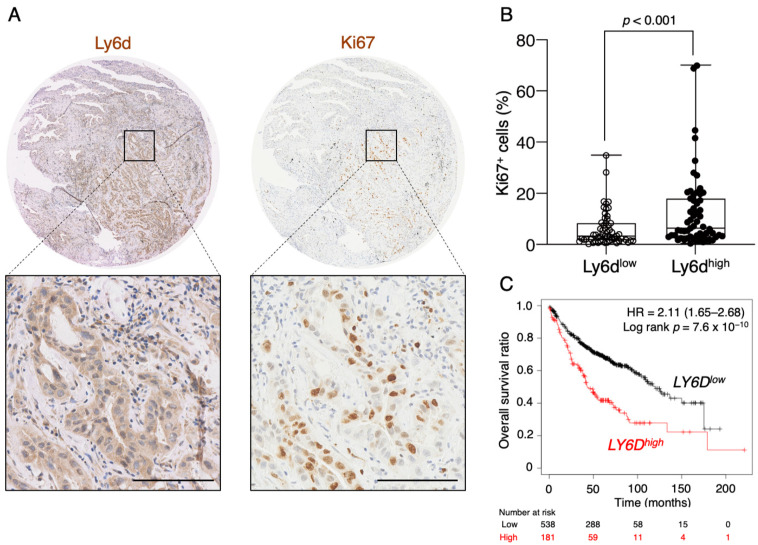
Expression of Ly6d is associated with poor prognosis in LUAD patients. (**A**) Representative immunohistochemical staining of Ly6d and Ki67 in a LUAD tumor sample. Scale bars, 50 µm. (**B**) Quantitation of the percentage of Ki67^+^ tumor cells per field in Ly6d^low^ (*n* = 60) and Ly6d^high^ (*n* = 60) LUAD tumors. Ly6d^low^ and Ly6d^high^ subpopulations of LUAD patients were classified based on the Ly6d expression levels with the mean values used for the cutoff points. Data are presented as box-and-whisker plots, with the boxes indicating the quartile values and the whiskers indicating minimum and maximum. The *p* value was calculated by Mann-Whitney test. (**C**) Kaplan-Meier analysis of overall survival according to *LY6D* gene expression in LUAD patients (*n* = 719) generated using Kaplan-Meier Plotter, with auto select cut-off selected. The hazard ratio (HR) with its 95% confidence interval as well as the log-rank *p* value are indicated.

To examine the possible effect of Ly6d expression on tumor aggressiveness in LUAD patients, we first performed immunohistochemical analysis of microarrays containing tumor tissue from 120 such individuals. We found that Ly6d^+^ tumor cells also expressed Ki67 (Figure 6A), which is a marker of cell proliferation and whose expression is associated with a poor outcome in lung cancer [47]. Furthermore, Ki67^+^ cells were observed more frequently in tumors with a high expression level of Ly6d than in those with a low level (Figure 6B). Finally, analysis of the relation between *LY6D* gene expression and overall survival in 719 LUAD patients revealed that a high expression level was significantly associated with a poor overall survival (Figure 6C). These results together thus suggested that Ly6d plays an important role in tumor cell proliferation in LUAD and thereby promotes tumor aggressiveness.

## 3. Discussion

Somatic mutations of *EGFR* and *KRAS* as well as chromosome rearrangements affecting *ALK*, *ROS1*, and *RET* have been identified in human LUAD. To better understand the relation between such genetic alterations and the characteristics of lung cancer, we have developed organoid-based orthotopic and syngeneic mouse models of LUAD. EpCAM-positive lung epithelial cells isolated from *Cdkn2a* knockout mice were cultured as organoids to maintain epithelial stem cell properties. The cells were then transformed by KRAS(G12V) or EML4-ALK, giving rise to KC and AC cells, respectively. Both KC and AC cells formed tumors in bleomycin-pretreated lungs of wild-type syngeneic mice after intratracheal transplantation. The developed tumors expressed the lung lineage marker TTF-1 and closely recapitulated the pathology of human LUAD. Treatment with crizotinib at a low concentration suppressed the growth of the *EML4-ALK*–transformed (AC) tumor cells, but not those of the KRAS(G12V)-expressing (KC) tumor cells. The proliferation of KC and AC cells appears to be highly dependent on their oncogenes, as is the case for human LUAD [35,36]. In addition, crizotinib treatment for 14 days induced complete regression of tumors formed in our AC models, whereas the tumors regrew after discontinuation of the drug. Our LUAD mouse models should prove useful for studies of the pathogenesis, treatment, and recurrence of lung cancer.

The recent development of 3D culture methods has allowed prolonged culture of epithelial cells from various organs including the lung [48]. Feeder-free 3D culture was initially established for intestinal stem cells [49,50] and was subsequently applied to various endodermal organs [51,52]. We established a feeder-free 3D culture method for lung epithelial cells based on Matrigel and serum-free medium supplemented with EGF, KGF, and a ROCK inhibitor. In such culture, EpCAM^+^CD31^−^CD45^−^ lung epithelial cells isolated from C57BL/6 mice gave rise to three types of colonies—cystic (resembling bronchi), spherical, and branched (resembling alveoli)—that contained cells that expressed lung differentiation markers. The lungs are composed of various differentiated epithelial cell types—including club cells as well as type I and type II alveolar cells—and EpCAM is widely expressed in these cells. The observed differences in colony morphology may result from differences in the cells of origin or differentiation lineages. However, serial passage resulted in enrichment of sphere-type colonies, with most of the cells in these colonies no longer expressing differentiation markers including SP-C and CC-10 but were positive for EpCAM, SCA-1, and TTF-1. These cells appeared to adopt an undifferentiated state while retaining the lung epithelial cell lineage after passage. Previous observations [53,54] suggest the possibility that the ROCK inhibitor present in the culture medium was responsible for conversion of the differentiated epithelial cells to an undifferentiated state. It is also possible that the differentiated cells die and that a minor population of undifferentiated stemlike cells with the ability to self-renew subsequently expands. Further studies will be necessary to distinguish between these and other possibilities. Although EpCAM^+^SCA-1^+^ lung cells had been thought to be BASCs [24,25], few SP-C and CC-10 double-positive cells were detected in our cultures after passage despite their EpCAM^+^SCA-1^+^ phenotype. This finding is consistent with a recent study showing that SCA-1 is not a marker for mouse BASCs that can be traced to cells co-expressing SP-C and CC-10 [55]. Most of the cells in our culture system after passage may therefore belong to a lung stem cell population other than BASCs.

Our 3D culture condition enriched EpCAM^+^SCA-1(Ly6a)^+^ cells as well as another member of the Ly6/uPAR family, Ly6d, was enriched by 3D culture. In this study, Ly6d was found to be required for the growth of LUAD-initiating (KC and AC) cells, suggesting that Ly6d is a potential CSC marker. Ly6d has previously been identified as a marker for progenitor cells in some organs [44,45,46]. We found that Ly6d and SCA-1 show similar expression patterns in our model system, and our observation that knockdown of *Ly6d* expression significantly attenuated the colony-forming ability of KC and AC cells suggested that Ly6d plays a key role in the self-renewal of tumor cells. We also found that human LUAD tumors with a high level of Ly6d protein expression harbored more Ki67-positive proliferating cells than did those with a low level of Ly6d expression. Additionally, a high level of *LY6D* mRNA was associated with a poor overall survival in LUAD patients, further implicating Ly6d as a potential CSC marker.

Although most of the cultured KC and AC cells expressed SCA-1, which is a marker for CSCs [38,39,40] before transplantation, these cells formed tumors consisting of SCA-1^+^ and SCA-1^−^ tumor cells, showing the heterogeneous patterns of SCA-1 expression in mice. The SCA-1^−^ tumor cells might be derived from SCA-1^+^ CSCs. CSCs are involved in the generation of intra-tumoral heterogeneity [56], and the TME is thought to contribute to the maintenance of CSC stemness as well as to the development of intra-tumoral heterogeneity [57,58,59]. Our results suggest that SCA-1^+^ KC or AC cells possess undifferentiated stem cell characteristics, and that the TME affect KC and AC cells leading to the development of heterogeneous tumors in mice.

Unlike previous studies of orthotopic transplantation of tumor cells into the mouse lung, including in syngeneic animals [30,31], the recipient C57BL/6 mice in this present study required bleomycin pretreatment for establishing lung tumors in both KC and AC models. One possible explanation for this difference is that the expression of GFP in KC and AC cells may have conferred immunogenicity in lungs and promoted tumor cell rejection by the recipient mice [60]. Bleomycin-induced lung fibrosis is accompanied by increased infiltration of immunosuppressant cells such as regulatory T cells [61] and M2-type macrophages [62], and these cells may mitigate rejection of the transplanted tumor cells. Activated fibroblasts in fibrotic foci can also support and maintain the stemness of engrafted tumor cells [63]. Bleomycin-induced lung fibrosis is transient, and we indeed observed negligible remnants of fibrosis in the lungs of our KC and AC models. Together with CSCs, the TME is therefore also an important therapeutic target. Indeed, anticancer therapeutic strategies that target nontumor cells—in particular, immune checkpoint blockade—have been proven to be effective [64,65]. Given that they are based on immunocompetent syngeneic mice, our LUAD models will be helpful tools for investing crosstalk between CSCs and the TME.

Our results show that mouse lung epithelial stem cells were enriched under our 3D culture conditions and that deletion of *Cdkn2a* and the introduction of mutant *KRAS* or *EML4-ALK* transformed these cells into LUAD-initiating cells that are capable of forming human LUAD–like tumors in syngeneic mouse lungs. In addition, we identified Ly6d as a functional CSC marker of LUAD. Ly6d has a potential to provide the basis for developing novel LUAD therapeutic strategies.

## 4. Materials and Methods

### 4.1. Mice

Six- to 10-week-old C57BL/6J wild-type mice (Sankyo Labo Service Corporation, Tokyo, Japan), *Cdkn2a*^−/−^ mice (B6.129-*Cdkn2a^tm1Rdp^*; National Cancer Institute, Frederick, MD, USA), and nude mice (CAnN.Cg-*Foxn1^nu^*/CrlCrlj) (CLEA Japan, Tokyo, Japan) were used for this study. All animal procedures were conducted under guidelines approved by the ethics committee of Keio University School of Medicine (approval number 15019).

### 4.2. Isolation of Lung Epithelial Cells from Mice

C57BL/6 wild-type mice or *Cdkn2a*^−/−^ mice were overdosed with intraperitoneal pentobarbital (Kyoritsu Seiyaku, Tokyo, Japan) and then subjected to exsanguination by severance of the abdominal aorta. The lungs were exposed and perfused via the right atrium with 10 to 15 mL of ice-cold PBS with the use of a 20-mL syringe fitted with a 23G needle. A 1.5-mL volume of collagenase-dispase (Roche, Basel, Switzerland) solution (2.5 mg/mL), followed by 0.5 mL of melted agarose (Nacalai Tesque, Kyoto, Japan), was then introduced into the lungs via the trachea. The lungs were covered with ice for 2 min to promote solidification of the agarose, after which the lung lobes were transferred to a tube containing collagenase-dispase (2 mL per mouse) and incubated for 10 min in a water bath at 37 °C. The lung tissue was minced into small pieces and further incubated in collagenase-dispase solution at 37 °C on a rotating platform for 45 min. DNase I (Sigma, St. Louis, MO, USA) was added to a final concentration of 500 µg/mL, and the suspension was passed first through the tip of a 1000-µL pipette and then through consecutive filters with pore sizes of 100 and 40 µm. The final filtrate was centrifuged at 200× *g* for 3 min at 4 °C, and the resulting pellet was suspended in 10 mL of erythrocyte lysis buffer (154 mM NH_4_Cl, 14.1 mM NaHCO_3_, 0.13 mM EDTA) and incubated for 1 min at room temperature. The suspension was centrifuged again, and the new pellet was suspended in 1 mL of wash buffer (PBS supplemented with 1% fetal bovine serum and 15 mM sodium azide). Cells were stained on a rotating platform for 20 min at 4 °C with the following antibodies: phycoerythrin (PE)–conjugated anti–mouse CD45 (30-F11; eBioscience, San Diego, CA, USA), PE-conjugated anti–mouse CD31 (390; BioLegend, San Diego, CA, USA), and allophycocyanin (APC)–conjugated anti–mouse EpCAM (G8.8, BioLegend). EpCAM^+^CD45^−^CD31^−^ cells were isolated by FACS (Figure 1B) with a MoFlo XDP Cell Sorter (Beckman Coulter, Brea, CA, USA).

### 4.3. Culture of Lung Cells

Cell culture inserts for 24-well plates (pore size of 0.4 µm; Corning, Corning, NY, USA) were coated with 50 µL of growth factor–reduced Matrigel (Corning) before cell seeding. After sorting, cells were isolated by centrifugation and suspended in lung cell medium (LCM) consisting of Dulbecco’s modified Eagle’s medium (DMEM)–nutrient mixture F-12 (Wako, Osaka, Japan) supplemented with B-27 (Thermo Fisher Scientific, Waltham, MA, USA), 15 mM HEPES (Thermo Fisher Scientific), as well as recombinant murine EGF (40 ng/mL, Peprotech, Cranbury, NJ, USA), recombinant murine KGF (20 ng/mL, Peprotech), and 10 µM of the ROCK inhibitor Y-27632 (Millipore, Burlington, MA, USA). The cells were then seeded at a density of 1 × 10^5^ cells/mL in 200 µL on each insert of a 24-well plate, 400 µL of LCM were added to the bottom of each well, and the medium in both chambers was replaced every 2 to 3 days. Colonies were observed and were counted after 10 days with the use of a BZ-9000 microscope (Keyence, Osaka, Japan).

### 4.4. Retrovirus-Mediated Oncogene Transduction

Full-length human *EML4-ALK* and *KRAS*^G12V^ cDNAs were amplified by the polymerase chain reaction (PCR) from the plasmids pcDNA-EML_ALK-V5#9 and pGCDN-KRAS^G12V^-IRES-huKO^40^, respectively, with the primers EML4-ALK-sense (5′-ACGCGTCGACATGGACGGTTTCGCCGGCA GTCTCGATGA-3′, SalI site underlined), EML4-ALK-antisense (5′-ATAAGAATGCGGCCGCTTAG GGCCCAGGCTGGTTCATGC-3′, NotI site underlined), KRAS^G12V^-sense (5′-CGGGATCCATGACTG AATATAAACTTGTGGTAGTTGGAGC-3′, BamHI site underlined), and KRAS^G12V^-antisense (5′-ATAAGAATGCGGCCGCTTACATAATTACACA CTTTGTC-3′, NotI site underlined). The PCR products were digested with the corresponding restriction enzymes and then ligated into the pMXs-IRES-GFP retroviral vector plasmid [66] to yield pMXs-IG-EML4-ALK and pMXs-IG-KRAS^G12V^, respectively.

For retrovirus production, 4 × 10^6^ GP2-293 cells (Takara, Tokyo, Japan) were transferred in 10 mL of DMEM (Wako) supplemented with 10% fetal bovine serum into each of two 100-mm dishes for each type of oncogene virus on the morning of day 1. In the evening, the cells were transfected with the retroviral vector plasmid (pMXs-IG-KRAS^G12V^ or pMXs-IG-EML4-ALK) and pVSV-G with the use of the FuGENE HD reagent (Promega, Madison, WI, USA) in Opti-MEM (Thermo Fisher Scientific). After 5 to 10 h, the medium was replaced with fresh DMEM supplemented with 10% fetal bovine serum. On the evening of day 4, the virus-containing medium from each pair was collected, passed through a 0.45-µm filter into a 50-mL tube, and centrifuged at 23,000× *g* for 6 to 7 h at 4 °C, after which the virus pellet was suspended in 1.2 mL of LCM.

Lung cell colonies were dissociated by exposure to 0.5% trypsin-EDTA (Thermo Fisher Scientific) followed by filtration with a 40-μm nylon cell strainer. After the addition of trypsin neutralization solution (Lonza, Morristown, NJ, USA), the cells were isolated by centrifugation, mixed with the virus suspension at a density of 1 × 10^5^ cells per 200 µL, and transferred to the inserts of a 24-well plate (2 × 10^4^ cells per insert). LCM (400 µL) was added to the bottom of each well. At 12 to 14 days after the onset of infection, GFP-positive cells were sorted by FACS with a MoFlo XDP Cell Sorter.

### 4.5. Animal Experiments

For the orthotopic syngeneic mouse models, the recipient C57BL/6 wild-type mouse was anesthetized with 4 mg/kg of midazolam (Wako), 0.3 mg/kg of medetomidine (Wako), and 5 mg/kg of butorphanol (Wako), and placed on a tilted platform. A 50-µL volume of bleomycin (Tokyo Chemical Industry, Tokyo, Japan) at 0.5 mg/mL was administered to the mouse via the trachea with the use of a cannula. At 2 weeks after bleomycin treatment, a single-cell suspension of KC or AC cells 1 × 10^5^ cells in 50 µL of PBS was introduced into the lungs via the trachea as for bleomycin administration (Supplementary Figure S3, Supplementary Video S1). At the end of experiments, tumors were removed from mice and fixed in 4% paraformaldehyde (Wako) overnight.

For the subcutaneous injection model, a single-cell suspension of AC cells 1 × 10^6^ cells in 100 µL of 50% growth factor–reduced was injected under the skin on the dorsal flank of a nude mouse. Tumor volume (mm^3^) was calculated every 2 days according to the formula: short axis^2^ × long axis × 0.5236. When tumor size reached ~75 mm^3^, mice were randomly assigned to receive vehicle (Methyl cellulose, Wako) or crizotinib (Pfizer, New York, NY, USA) at 150 mg/kg by daily oral administration.

### 4.6. Histological Analysis and Immunostaining

Fixed tumor samples or normal lung tissue from mice were embedded in paraffin. Tumor tissue microarrays derived from LUAD patients were obtained from US Biomax (Derwood, MD, USA). Cultured organoids were immobilized with iPGell (GenoStaff, Tokyo, Japan) according to the manufacturer’s instruction. Organoids were then fixed with 4% paraformaldehyde for 30 min at room temperature and embedded in paraffin. Tissue sections or organoids were subjected to hematoxylin-eosin, immunochemical, or immunofluorescence staining. Immunochemical analysis was performed with antibodies to TTF-1 (EP1584Y; Abcam, Cambridge, UK), to SP-C (FL-197; Santa Cruz Biotechnology, Dallas, TX, USA), to CC-10 (T-18, Santa Cruz Biotechnology), to AQP5 (D-7, Santa Cruz Biotechnology), to GFP (FL, Santa Cruz Biotechnology), to Ly6d (17361-1-AP; Proteintech, Rosemont, IL, USA), and to Ki67 (SP6, Abcam), and immune complexes were detected with the use of a Vectastain Elite ABC-HRP Kit (Vector, Burlingame, CA, USA). Immunofluorescence analysis was performed with antibodies to SP-C (as above), to CC-10 (as above), to Ly6d (as above), and to SCA-1 (E13-161.7, BioLegend), with immune complexes being detected with Alexa Fluor 488– or Alexa Fluor 594–conjugated mouse or goat secondary antibodies (Thermo Fisher Scientific) and nuclei being stained with 4′,6-diamidino-2-phenylindole (DAPI). Tissue sections and organoids were observed with a BZ-9000 microscope or an FV1000-D confocal microscope (Olympus, Tokyo, Japan). Tissue microarrays were scanned with a NanoZoomer instrument (Hamamatsu Photonics, Shizuoka, Japan) and analyzed with QuPath software [67].

### 4.7. Flow Cytometry

Single-cell suspensions of cultured cells were stained for 30 min at 4 °C with the following antibodies: PE- and Cy7-conjugated anti–SCA-1 (E13-161.7, BioLegend), PE-conjugated anti–mouse Ly6d (49-H4, BioLegend), and APC-conjugated anti–mouse EpCAM (as above). Samples were analyzed with an Attune flow cytometer (Thermo Fisher Scientific) and with the use of appropriate single-color controls for compensation. Data were analyzed with Attune software.

### 4.8. Cell Viability Assay

KC or AC cells 1 × 10^4^ in 100 µL of LCM were plated in a 96-well plate that had been coated with growth factor–reduced Matrigel. The cells were cultured overnight before exposure to various concentrations of crizotinib for 72 h, after which cell viability was determined with the use of CellTiter-Glo (Promega).

### 4.9. RNA Interference

To introduce short hairpin RNAs into the cells, we used the lentiviral vector pLKO.1 (Sigma), which also contains puromycin resistance genes. The sequences of the sense oligonucleotides were 5′- CCGGGTAATGTACTGCTTGGAATTTCTCGAGAAATTCCAAG CAGTACATTACTTTTTTG-3′ (Sigma) for Ly6d shRNA #2 and 5 ′-CCGGGCACCAACAGTGCCAACTGTACTCGAGTACAGTTG GCACTGTTGGTGCTTTTTG-3′ (Sigma) for Ly6d shRNA #5. Lentiviral production and infection to KC or AC cells were performed as described in ‘Retrovirus-Mediated Oncogene Transduction’. At 12 to 14 days after the onset of lentivirus infection, KC or AC cells were subjected to selection in the presence of puromycin (5 mg/mL).

### 4.10. RT-qPCR Analysis

The expression of Ly6/uPAR family genes in primary and serially passaged mouse lung epithelial cells was measured by reverse transcription (RT) and quantitative PCR (qPCR) analysis. Total RNA was extracted from cells and purified with the use of the RNeasy Mini Kit (Qiagen, Venlo, The Netherlands), and cDNA was synthesized from 10 to 1000 ng of the RNA with the use of a Transcriptor First Strand cDNA Synthesis Kit (Roche, Basel, Switzerland). The cDNA was subjected to qPCR with the primers listed in Supplementary Table S1. The expression level of each gene was normalized by that of *Gapdh*, and hierarchical clustering was performed with the use of Gene Cluster 3.0 software.

### 4.11. Statistical Analysis

Statistical analysis and sample sizes for each experiment are described in the figure legends. Statistical analysis was performed with GraphPad Prism 7 (GraphPad Software, San Diego, CA, USA), with the exception that Kaplan-Meier Plotter (https://kmplot.com/analysis) [68] was used for survival analysis in LUAD patients based on *LY6D* expression.

## 5. Conclusions

In this study, we established mouse LUAD cells with tumor-initiating ability driven by loss of *Cdkn2a* and expression of mutant *KRAS* or *EML4-ALK* oncogenes. Orthotopic transplantation of these cells into syngeneic mouse lung resulted in the formation of tumors that mimicked human LUAD, with the rate of tumor formation being greatly increased by the induction of transient fibrosis by bleomycin administration. With the use of these models, we identified Ly6d as a potential novel CSC marker for LUAD. Given their clinical relevance, our mouse models should prove to be important tools for drug development and investigations of CSC biology in LUAD.

## 6. Patents

A patent application is pending for the method developed in this study (WO2018164174, PCT/JP2018/008735).

## Supplementary Materials

The following are available online at https://www.mdpi.com/2072-6694/12/12/3805/s1. Figure S1: The expressions of KRAS, ALK, and GFP. Figure S2: Ly6d expression in KC and AC cells. Figure S3: Procedure for intratracheal injection. Table S1: List of PCR primer sequences. Video S1: Technique for intratracheal intubation and bleomycin administration. Supplementary Materials and Methods: Immunoblot analysis.
