# Supplementary material for: Lung Adenocarcinoma Mouse Models Based on Orthotopic Transplantation of Syngeneic Tumor-Initiating Cells Expressing EpCAM, SCA-1, and Ly6d
Source: Cancers (Basel). 2020 Dec 17;12(12):3805. doi: 10.3390/cancers12123805 (PMC7767274; doi:10.3390/cancers12123805)
Supplement: Supplementary file 1 [file cancers-12-03805-s001.zip › cancers-1037925-supplementary/_revision_SembaCAN_supplementary materials_arima.pdf]

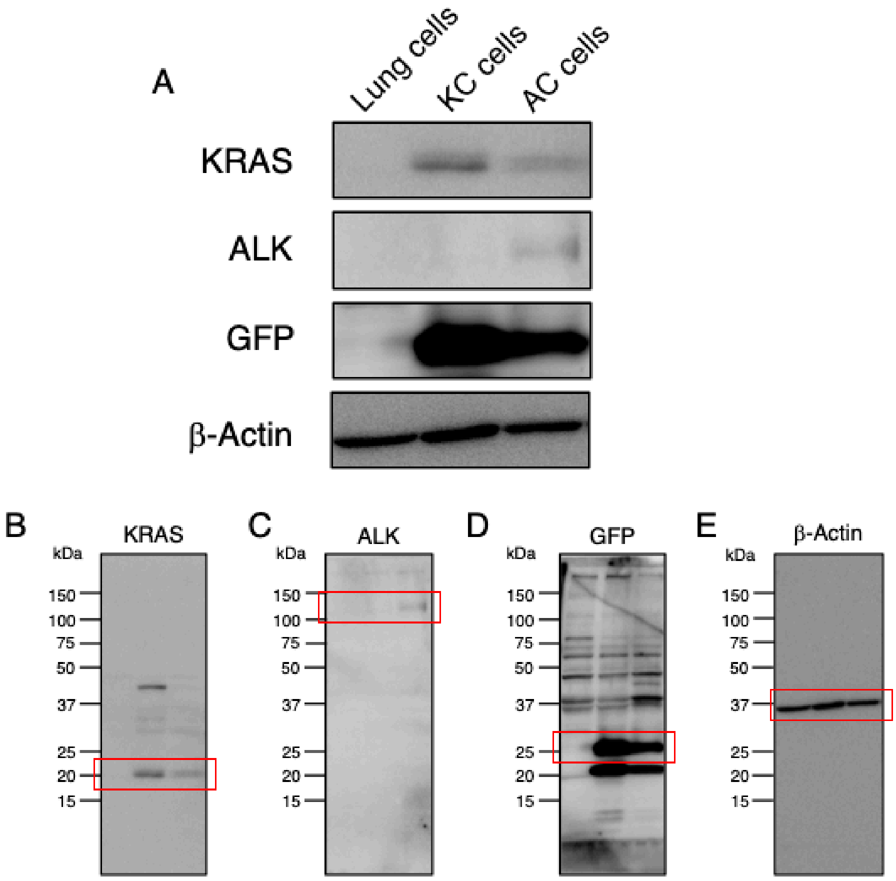

Supplementary Figure S1. The expressions of KRAS, ALK, and GFP. (**A**) Immunoblot analysis of KRAS, ALK, and GFP in cultured *Cdkn2a*$^{-/-}$ lung cells, KC, and AC cells. β-Actin was examined as a loading control. (**B** - **E**) The whole immunoblotting image for KRAS (**B**), ALK (**C**), GFP (**D**), and β-Actin (**E**).

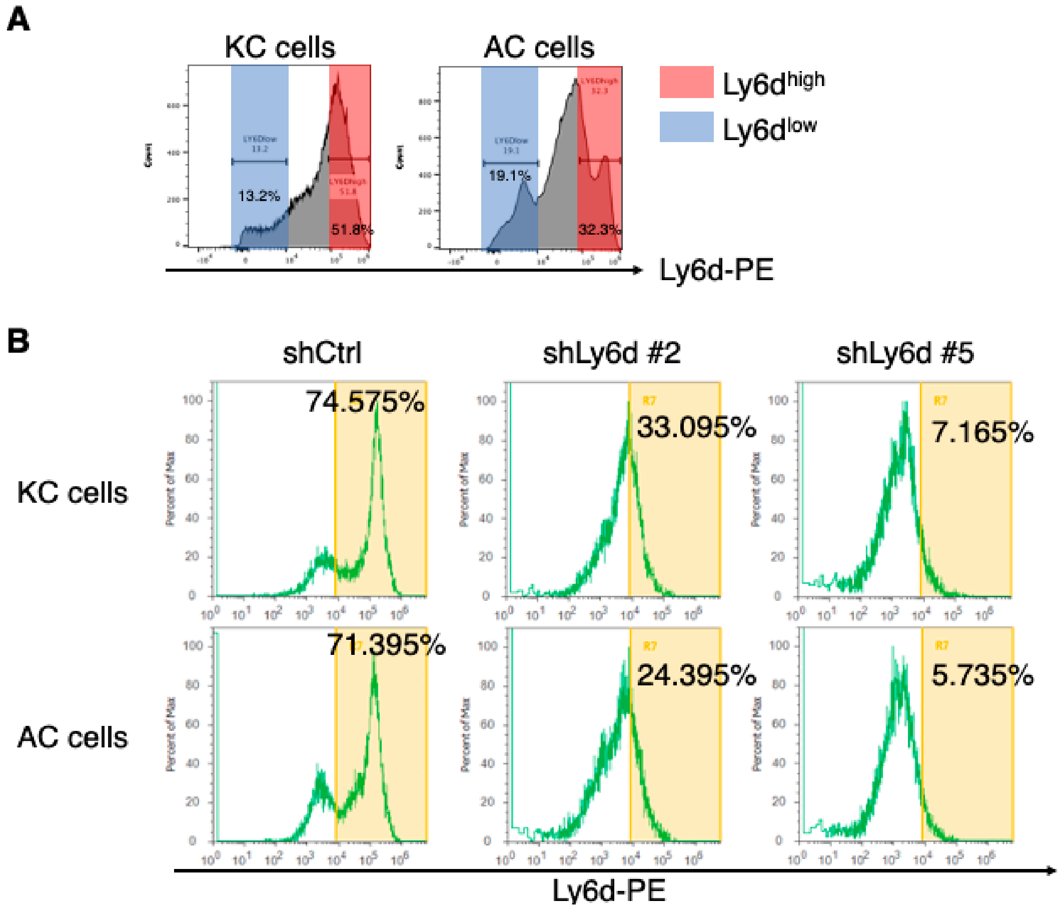

Supplementary Figure S2. Ly6d expression in KC and AC cells. (**A**) Gating strategy for isolation of Ly6d^{high} and Ly6d^{low} cells from KC and AC cells by FACS. (**B**) Validation of shRNA-mediated knockdown of Ly6d expression in KC and AC cells by flow cytometry.

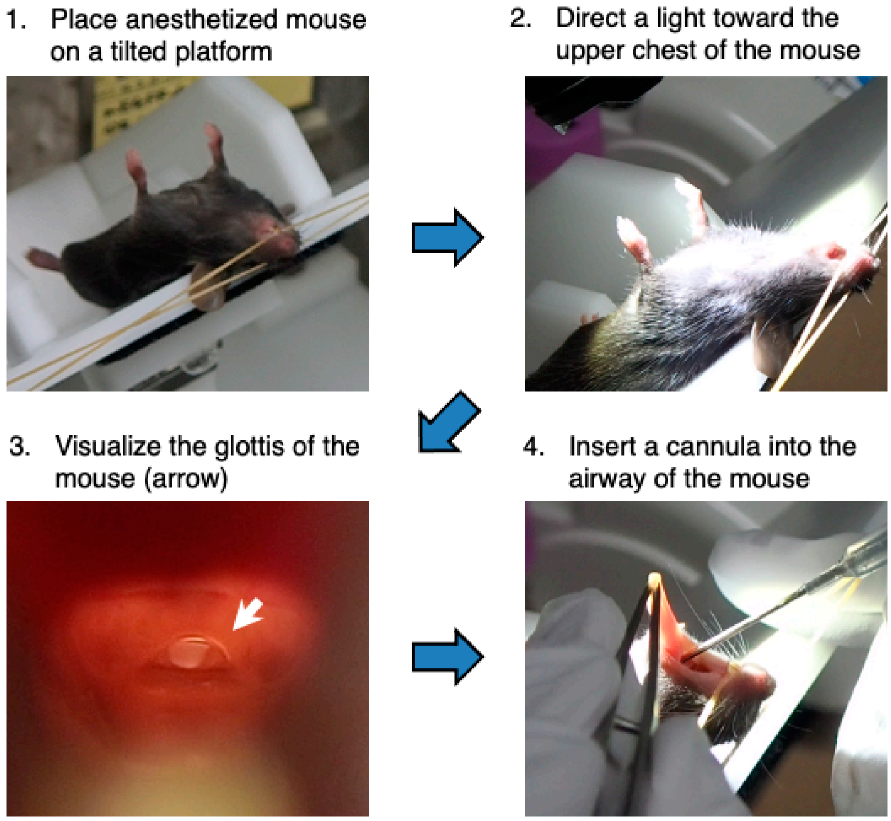

Supplementary Figure S3. Procedure for intratracheal injection.

Supplementary Table S1. List of PCR primer sequences. Fw, forward; Rv, reverse.

Gene symbol		Sequence (5' → 3')
Psca	Fw	TCATCTGTGCTGTGCATGAAT
	Rv	GCTCACTGCAACCATGAAGA
Slurp1	Fw	GTCACCATGGGACTGTGGTT
	Rv	TTGCTCAGTGCAAGATGGAA
Lypd2	Fw	GCATCCAACTGTGTCACCAC
	Rv	GTCAGAATTGCAGCAGGACA
Slurp2	Fw	TCCACCCACTGTGTCATCAT
	Rv	GGAGTGTGGTCAGTCCCTGT
Lynx1	Fw	TGCCTGAGCTCTTGGTCTCT
	Rv	TACCAACACCGCACGAAGT
Ly6d	Fw	AGACAGCTCTGCTCGTCCTC
	Rv	CTGTAGTCGGAGGTGCATGA
Ly6k	Fw	CAGGCATAGCTGTTCTGCG
	Rv	CCTAGTGGCCTTGCTCGTAG
Gml	Fw	TCTTGTGCTGTGGGAGTGTC
	Rv	CTGGAGAGGATCAAGGCAAG
Ly6e	Fw	ACAGCACAGGCAGGAAGACT
	Rv	TCGGTATTATCTTCGGGGC
Ly6a	Fw	GGCAGATGGGTAAGCAAAGA
	Rv	CAATTACCTGCCCCTACCCT
Ly6h	Fw	AATTGGCCAGGGTGCAGT
	Rv	ATGAAGAGCCTCGGTCTGG
Gpihbp1	Fw	CTGGAGCAGCTCTGTGTCTG
	Rv	ACCAACATGATCCCTGGAAG
Gapdh	Fw	GTGAAGGTCGGTGTGAACG
	Rv	GACCATGTAGTTGAGGTCAATG

Supplementary Materials and Methods

Immunoblot analysis

Cultured lung cells, KC and AC cells were collected by digesting Matrigel using Cell Dissociation Solution (Corning, Corning, NY, USA). Cells were lysed with SDS sample buffer (2% SDS, 10% glycerol, 50 mM Tris-HCl [pH 6.8]) containing 1 M DTT for fractionation by SDS-PAGE. The separated proteins were transferred to a polyvinylidene difluoride membrane and exposed to anti-KRAS antibody (C-19; Santa Cruz Biotechnology, Dallas, TX, USA), anti-ALK antibody (D5F3; Cell Signaling Technology, Danvers, MA, USA), anti-GFP antibody (FL, Santa Cruz Biotechnology), and anti-β-Actin antibody (sc-47778, Santa Cruz Biotechnology). Immune complexes were detected with horseradish peroxidase-conjugated anti-rabbit antibody (Agilent, Santa Clara, CA, USA) or anti-mouse antibody (Agilent) and enhanced chemiluminescence reagents (Chemi-Lumi One L, Nacalai Tesque, Kyoto, Japan).
